# Educational achievement at age 9.5 years of children born to mothers maintained on methadone during pregnancy

**DOI:** 10.1371/journal.pone.0223685

**Published:** 2019-10-10

**Authors:** Samantha J. Lee, Lianne J. Woodward, Jacqueline M. T. Henderson

**Affiliations:** 1 School of Psychology, Speech and Hearing, University of Canterbury, Christchurch, New Zealand; 2 School of Health Sciences, University of Canterbury, Christchurch, New Zealand; Medizinische Universitat Wien, AUSTRIA

## Abstract

Recent research shows that preschool children born to opioid-dependent mothers are at increased risk for cognitive, psychomotor, attention, and social-emotional adjustment problems. But very little is known about their school-age functioning, particularly their educational achievement. This analysis examined the educational outcomes of a regional cohort of 100 prenatally methadone-exposed children who were prospectively studied from birth to age 9.5 years alongside a comparison group of 110 randomly identified non-exposed children born between 2003 and 2008. At age 9.5, as part of a comprehensive neurodevelopmental evaluation, children’s teachers rated their achievement across the school curriculum, and children completed the Woodcock Johnson-III Tests of Achievement (WJ-III). Detailed information about the birth mother’s social background, pregnancy substance use, and mental health was also collected during pregnancy/at term. Infant clinical data were collected after birth. Methadone-exposed children performed less well than non-exposed children across seven school curriculum areas rated by teachers (*p*s ≤.001), performed less well than non-exposed children on all reading and mathematics subtests of the WJ-III, and had higher rates of any educational delay on the WJ-III (57% vs. 15%), OR = 7.47 (3.71–15.02). Results were similar when children with severe intellectual impairment were excluded. After adjusting for confounding factors, methadone-exposed children had increased odds of educational delay, but this was only marginally significant (OR = 3.62, [1.01–13.01], *p* = .049). Maternal educational attainment level (OR = 0.69, [0.50–0.89], p = .006), and maternal benzodiazepine use during pregnancy (OR = 2.70 [1.03–7.12], p = .044) were also associated with later educational risk. Findings suggest that children born to opioid-dependent women enrolled in methadone maintenance are at high risk of educational delay by age 9.5 years. Children’s academic difficulties appeared to reflect the effects of both adverse prenatal exposures and postnatal social risk.

## Introduction

The impact of the recent opioid epidemic on children and families is of growing concern, with recent data suggesting that the risks associated with maternal opioid use during pregnancy likely extend beyond neonatal abstinence syndrome (NAS) and other neonatal complications. For example, data from a 4 ½ year follow-up study found that, at school entry, children born to opioid-dependent mothers were at increased risk for a range of health and neurodevelopmental difficulties [1]. These spanned behavioural, attention, visual-motor, and cognitive domains. Although the mechanisms that account for these adverse outcomes are likely to be complex, these early findings do raise serious concerns about the possible long term challenges that these children will face, especially as they transition to school and the demands of the classroom [2, 3]. Yet almost nothing is known about the school-age functioning, especially the educational achievement, of children born to opioid-dependent mothers.

During pregnancy, methadone maintenance treatment (MMT) is the most common treatment approach for opioid-dependent women. At optimal doses, it reduces opioid craving and withdrawal, thus reducing the risk of relapse to illicit opioid use [4]. MMT is also associated with a reduced risk of negative maternal and infant health outcomes linked with maternal illicit opioid abuse and which have significant impacts on maternal and infant well-being and health service use [5–8]. But nonetheless, methadone does cross the placenta during pregnancy [9, 10] resulting in high rates (45% to >90%) of NAS (also referred to as Neonatal Opioid Withdrawal Syndrome or NOWS) after birth [11, 12]. Preterm birth and intrauterine growth restriction are also more common [11, 13, 14], and have been linked with later cognitive and learning difficulties [15–17]. There is also growing suggestion that prenatal exposure to opioids may have adverse effects on the developing brain that persist into childhood. Term born neonates whose mothers used opioids during pregnancy are characterized by reduced whole brain and basal ganglia volumes compared to general population means at birth [18]. Microstructural alterations in white matter tracts have also been observed [19, 20]. But even more importantly, several small-scale studies suggest that these neurological changes continue to be observed at school age [21, 22, 23], reinforcing existing concerns about the potential long term cognitive and educational outcomes for these children.

In addition to possible neurological impacts, methadone-exposed (ME) children are also more likely to be raised in psychosocially adverse and chaotic home environments when compared to children born to non-drug-dependent mothers, with these family experiences compounding educational risks. Studies have shown that maternal opioid dependence tends to occur in the context of multiple psychosocial risk factors that include educational underachievement, financial instability, high rates of unemployment and single parenthood, and ongoing maternal substance use and comorbid mental health problems [24–27].

To date, only one small-scale, and somewhat dated (25-year-old), retrospective study has assessed the reading and mathematics achievement of school-age children born to mothers maintained on methadone during pregnancy [28]. They found no differences between ME and non-ME children on a standardized achievement measure. However several limitations suggest these results should be interpreted cautiously. Importantly, the small sample size (n = 20) limited the statistical power to detect between-group effects. The exposed and comparison groups were also matched for low socioeconomic status (SES) and neonatal risk (preterm birth, growth restriction) which, whilst helpful in controlling for background risk factors, does make it difficult to accurately determine the full extent of ME children’s educational risk relative to the general population.

Although a better understanding of the educational needs of children born to mothers who were maintained on methadone during pregnancy is clearly needed, findings from other studies of opioid-exposed groups lend further support to concerns about the potential poorer academic achievement of these children. A population data linkage study by Oei et al. (2017) examined the school curriculum-based achievement of children with a NAS diagnosis [29] and found that across middle childhood, they obtained poorer numeracy and literacy scores than demographically matched children without NAS. Associations between NAS and later educational delay remained significant after covariate adjustment for infant (male, preterm birth) and socio-familial factors (young motherhood, low parental educational attainment, ethnic minority) [29]. However, due to the reliance on medical records to identify the participants in this study, the authors were unable to accurately take account of the extent of other illicit substances during pregnancy. Also unclear was the extent to which children were subject to co-occurring or pervasive educational problems. Importantly, reading and mathematics difficulties often co-occur, and children who are affected in both of these academic domains typically have a more problematic educational trajectory than children with difficulty in one, or neither, domains [30–32].

Oei and colleagues’ findings are in some agreement with the results of Ornoy et al., (2001), who found large differences between the standardized reading and mathematics test performance of children who were born to heroin-addicted mothers, and a group of control children from average SES families [33]. Of interest, smaller differences in reading and mathematics scores were found when exposed children who were adopted into average SES families were compared with the controls, indicating that growing up in a more advantaged family environment may potentially help ameliorate some of the negative impacts of prenatal opioid exposure on children’s educational achievement. However, this study was also limited by a small sample, retrospective design, and no adjustment for covariate factors.

Against this background, the key aims of the current study were:

1. To describe the educational achievement of a cohort of 9.5-year-old ME children compared to a regionally representative non-ME group. Educational achievement was assessed using a multi-method approach that included teacher report and independent standardized educational testing.
2. To assess the extent to which any between-group differences in educational achievement reflected the direct effects of prenatal methadone exposure after adjustment for confounding factors spanning other prenatal substance exposures, infant medical risks, and maternal social background factors.

## Materials and methods

### Study design and participants

This study draws data from a prospective longitudinal study of two groups of children born in Christchurch, New Zealand. All mothers were recruited during their third trimester of pregnancy, or at birth, between 2003 and 2008. Exclusion criteria across both groups included very preterm birth (≤32 weeks), congenital abnormality, HIV diagnosis, suspected fetal alcohol syndrome, intent to deliver outside the region, maternal inability to provide informed consent, and non-English speaking mother. A detailed description of the study cohort recruitment procedure is provided in an earlier publication [25].

#### Methadone-exposed group

The first group comprised 100 children who were born consecutively to opioid-dependent mothers enrolled in MMT during pregnancy. All ME infants were born at Christchurch Women’s Hospital, the largest maternity care provider in the Canterbury region of New Zealand. The 100 ME infants and their mothers who were successfully recruited to the study represented 83% of all ME infants born in the Canterbury region during that time period. There were 85 ME child-caregiver dyads who participated in the study at age 9.5 years (85% retention). Reasons for participant loss to age 9.5 included declined participation (n = 11) and infant death (n = 4). One additional child could not complete the educational measures due to severe neurodevelopmental delay, and therefore this paper reports educational achievement data for 84 ME children. There were no differences between the participants and non-participants on any maternal psychosocial or infant clinical characteristics.

#### Non-exposed comparison group

The second group comprised 110 non-ME children whose mothers were identified at random from the delivery schedule of Christchurch Women’s Hospital. Control participants were born during the same time period and matched for expected birth date with children in the ME group (65% recruitment rate). Comparison of the socioeconomic profile of the recruited comparison families with regional census data showed that they were representative of families living in the region [34]. At age 9.5, 99 non-ME child-caregiver dyads were followed up (90% retention). Reasons for participant loss to age 9.5 years included declined participation (n = 5), relocation overseas (n = 3) and were subsequently excluded from analysis due to congenital anomaly or neurological disorder and severe neurodevelopmental delay (n = 3). There was a significantly higher proportion of children with mothers who had no educational qualifications among the non-participants, compared to the participants (*p* = .004).

### Procedure

Current study data were drawn from two time points: late pregnancy/birth and at age 9.5 years. During the third pregnancy trimester or at birth, all mothers provided informed consent to participate in the study, and a senior research nurse specialist administered a maternal psychosocial interview about each woman’s personal background, pregnancy nutrition, mental health, substance use/dependence, and current social and economic circumstances. In addition, maternal urine samples were collected at random across pregnancy, and infant meconium samples were collected at birth. Approximately 6 weeks following the child’s 9^th^ birthday, all children and their primary caregivers were invited to attend a comprehensive neurodevelopmental follow-up assessment that included standardized reading and mathematics achievement tests. All measures were administered in a laboratory setting by clinical research staff and graduate students who were blinded to the children’s group status where possible. Children’s classroom teachers were then sent a questionnaire about their school achievement and special education involvement. Teachers were not informed about the design of the study, and were therefore blinded to the child’s prenatal history. Ethical approval for the current study measures and procedures was obtained from the Southern Health and Disability Ethics Committee (*Reference*: URB/07/10/042). Caregivers who participated in the study provided written informed consent, and children who participated provided oral assent. Below is a description of the educational outcome measures at age 9.5 years, followed by a description of the covariate measures collected during pregnancy/at birth.

### Measures

#### Educational achievement at 9.5 years

**Teacher ratings of achievement**. Teachers were asked to rate each child’s achievement across eight curriculum domains including reading, mathematics, written language, spoken language, art, physical education, health, and technology [35]. For each domain, teachers indicated whether the child was: (1) more than 12-months delayed, (2) below average, (3) average, (4) above average, or (5) more than 12-months ahead relative to their classroom peers. For the current study we created a dichotomous variable indicating whether or not a child had delayed school performance, i.e. whether they were achieving either below average or more than 12-months delayed vs. those performing average to more than 12-months ahead, in each curriculum area.

**Standardized achievement tests**. Children completed six subtests from the standardized Woodcock Johnson-III Tests of Achievement (WJ-III) Form B, Australian adaptation [36]. The Broad Reading subtests were Letter-Word Identification, Reading Fluency, and Passage Comprehension, to measure children’s reading decoding skills, speed and accuracy, and reading understanding. The Broad Math subtests were Calculation, Math Fluency, and Applied Problem Solving, to measure children’s mathematical computation skills, speed and accuracy in solving basic operations, and problem solving ability. The six achievement subtests have strong internal consistency reliabilities ranging from α = 0.83 ‒ 0.92 [36]. The WJ-III also has good concurrent validity, correlating well with other validated achievement tests [37].

**Educational delay**. We defined an educational delay in reading and mathematics as a WJ-III score –1 SD below the comparison group mean. This corresponds to around a 12-month achievement delay, which is when, regardless of their general intellectual ability (IQ), children are considered for enrolment in special education services in New Zealand. This cut-point is also commonly used to define impairment amongst at-risk groups compared to their typically developing peers [16, 38]. To examine delay severity, children with WJ-III scores from –1 to –2 SDs below the comparison group mean were classified as having a mild delay, whereas those with scores more than –2 SDs lower than the comparison mean were classified as having a severe delay.

**Specific learning delay**. We examined rates of specific reading or mathematics delay using a low achievement criterion. Children with a specific reading or mathematics delay were those with a WJ-III score –1 SD below the comparison group mean that was not due to severe intellectual impairment. Children with severe intellectual impairment were identified in the current study using a cut-point of –2 SD below the comparison group mean on the Wechsler Abbreviated Scale of Intelligence–Second Edition (WASI-II) [39]. The WASI-II consists of four subtests that assess perceptual reasoning (Block Design, Matrix Reasoning) and verbal comprehension (Vocabulary, Similarities) to provide an estimation of each child’s IQ. These subtests have excellent internal consistency coefficients exceeding .90, with average internal consistency and test-retest reliability coefficients of greater than .92 for the total IQ estimate [40, 41]. The WASI-II has good concurrent validity, correlating well (.91 to .92) with other validated full-length intelligence measures [40]. There were 18 ME children and 2 non-ME children who met criteria for a severe intellectual impairment (IQ score ≤80), and were therefore excluded from the specific learning delay (SLD) analysis.

**Special education**. Both caregivers and teachers were questioned about each child’s access and use of special education assistance at school (e.g. teacher aide, individual education plan). For eligible children, special education is free in New Zealand. In the first instance, learning and/or behaviour support is provided by an in-class teacher aide on a priority-needs basis. Two other higher-tiered government-funded services are also available to 2% and 1% of the New Zealand school population with moderate/severe learning and/or behaviour problems, or exceptionally high learning needs and other behavioural/physical problems, respectively. New Zealand’s inclusive education system prioritises children remaining in their mainstream classrooms to continue learning alongside their typically developing peers. Therefore, it is very rare for children to repeat a grade/school year or be placed in a special school/classroom.

#### Covariate measures

**Maternal pregnancy substance use**. Maternal substance use was determined through (1) maternal interview, (2) random maternal urine sampling throughout pregnancy, and (3) infant meconium sampling after birth. First, methadone-maintained and comparison mothers were interviewed in confidence during their third trimester/at term about their substance use and dependence across pregnancy. Detailed information was obtained regarding the frequency and amount of prenatal tobacco, alcohol, cannabis, benzodiazepine, anti-depressant, stimulant and additional opioid use during each pregnancy trimester. Use of the latter five substances was confirmed through random maternal urinary drug screens that were obtained over the course of pregnancy for the mothers enrolled in MMT only, and through meconium samples that were obtained from most ME infants (81%) and close to half of the comparison infants at birth. The extent of children’s prenatal polysubstance exposure was also estimated from the three independent measures of maternal substance use during pregnancy. This polysubstance use score reflects the extent of maternal substance use (number and quantity of different substances used) during pregnancy, as previously described [1].

**Maternal social risk**. All mothers completed a comprehensive maternal lifestyles interview during their third trimester of pregnancy or at birth. Five maternal social risk indicators were obtained. These included maternal age, partner status (single, partner not cohabiting, partner cohabiting, married), ethnicity (New Zealand European, Māori, Pacific Islander, Asian or African), educational attainment level (1 to 6, with 1 = left school before age 16 with no qualifications, 2 = any secondary qualification [i.e. completed US 10^th^ or 11^th^ grade], 3 = further secondary qualification [i.e. US high school diploma], 4 = secretarial/trade qualification, 5 = professional qualification without a degree, and 6 = university degree), and socioeconomic status (1 to 7, with 1–2 = professional/managerial, 3–4 = technical/skilled, 5–6 = semi- and unskilled work, and 7 = unemployed) [42]. These variables were considered both individually, and as a composite social risk index [1], where each variable was dichotomized and summed so that higher scores indicated greater social risk.

**Maternal depression**. Maternal depression was assessed during the third trimester or at birth using the Edinburgh Postnatal Depression Scale (EPDS) [43]. The EPDS is a 10-item questionnaire, with statements including “I have felt sad or miserable” and “I have been so unhappy that I have been crying”. Statements were rated by participants on a 4-point scale, with reference to their depressive symptomology over the past 2 weeks. Higher total scores indicated greater depressive symptomology. A cut-off score of ≥13 on the EPDS is reported to have adequate sensitivity (79%) and specificity (85%) for identifying depression [44].

**Infant clinical data**. These data were obtained from hospital records and included infant sex, gestational age, birth weight, birth length, and birth head circumference. The birth weight, length, and head circumference measurements were then transformed to z-scores that adjusted for infant sex and gestational age.

### Statistical analyses

Analyses were performed using SPSS v.25. T-tests and chi square tests for independence were used to examine between-group differences in each educational outcome, with Cohen’s *d* or odds ratios giving a measure of effect size. Confidence intervals for Cohen’s *d* were calculated using ESCI [45]. This analysis was done including and excluding children with severe intellectual impairment to assess the extent to which any observed group differences might reflect the influence of these high risk children. Sex differences in children’s educational achievement, independent of study group, were examined using logistic regression for teacher ratings of achievement and two-way between-groups analysis of variance for children’s WJ-III scores. Interaction effects between study group and sex were also examined. Finally, logistic regression analysis was performed to examine the extent to which educational delay at age 9.5 years was associated with prenatal methadone exposure and/or associated prenatal substance exposures, infant medical risks, and maternal social background factors. A confounder was initially selected for use in the regression model if: (a) there was a significant between-group difference on that variable indicating its association with maternal enrolment in MMT during pregnancy (see Table 1), and/or (b) based on previous research and theory linking the confounder to children’s educational achievement. Variables that were significantly correlated with educational delay (*p* < .05) were included in the models. These were maternal educational attainment level, any maternal use of cigarettes, benzodiazepines, alcohol, or cannabis during pregnancy, and maternal depression score at term. Maternal SES was also associated with children’s educational delay. However, because of its high correlation with both methadone status and maternal educational achievement, and the greater theoretical importance of maternal education for child school achievement, this variable was selected for inclusion in the model over family SES. There were no significant correlations between educational delay and any of the infant clinical factors, and so these were not regressed in the final model. Finally, we assessed whether there were any interaction effects between group status and the significant covariates in the model.

**Table 1:** Sample characteristics.

	Methadone (*N =* 85)	Comparison (*N* = 99)	*p*
Maternal prenatal substance use			
*M (SD)* third trimester methadone dose	64.49 (32.40)	-	-
*M (SD)* polysubstance use score	6.02 (2.44)	0.80 (1.11)	< .001
% any cigarette use	91	16	< .001
% any cannabis use	52	1	< .001
% any benzodiazepine use	53	1	< .001
% any anti-depressant use	33	25	0.33
% any alcohol use	21	19	0.88
% any illicit opioid use	26	-	-
% any stimulant use	21	-	-
Maternal social background at term			
*M (SD)* total Social Risk score	2.56 (0.96)	0.76 (1.23)	< .001
% low family SES	93	24	< .001
% left school without qualifications	82	17	< .001
% single parent (not married or cohabiting)	52	10	< .001
% young mother (< 21 years)	4	5	0.89
% ethnic minority	26	19	0.28
*M (SD)* maternal depression score at term	12.04 (6.48)	5.11 (4.77)	< .001
Infant characteristics			
*M* (*SD*) birth weight z-score	-0.45 (0.75)	0.15 (0.91)	< .001
*M* (*SD*) birth length z-score	0.21 (1.14)	0.95 (1.12)	< .001
*M* (*SD*) birth head circumference z-score	-0.31 (0.88)	0.22 (0.92)	< .001
*M* (*SD*) gestational age, weeks	38.84 (1.7)	39.19 (1.7)	0.16
% preterm (<36 weeks)	9	8	0.96
% NAS treatment	87	-	-
% male sex	58	47	0.17
Caregiver characteristics at 9.5 year assessment			
% biological mother was primary caregiver	54	99	< .001
*M (SD)* caregiver age	45.28 (10.30)	41.41 (5.40)	.001
% low family SES	84	19	< .001
% single parent (not married or cohabiting)	60	13	< .001
% ethnic minority	18	21	0.57
Child characteristics			
*M* (*SD*) age at 9.5-year assessment	9.64 (0.40)	9.51 (0.34)	.02
*M (SD)* IQ score at age 9.5 years	93.83 (14.35)	108.33 (13.88)	< .001

## Results

### Sample characteristics

Table 1 describes the family social background, pregnancy exposures, and infant medical characteristics of the two study groups. Compared with infants born to non-opioid-dependent mothers, the mothers of ME children were more likely to engage in polysubstance use/abuse during their pregnancies, with their scores indicating that they more frequently used a wider range of different substances than comparison group mothers. Children born to mothers in MMT were also characterized by higher levels of family social risk, with the majority born into a lower SES family where their mothers had left school before age 16, and where they were being raised in a single parent family. There was no group difference in maternal age at birth or minority ethnic background (Māori, Pacific Islander, African, and Asian). ME and non-ME infants had a similar mean gestational age at birth, however ME infants were born smaller for their gestational age and sex, as indicated by their lower birth weight, length, and head circumference z scores. In total, 87% of ME infants were treated pharmacologically for NAS. There was no difference in the proportion of male and female children in each study group.

At the age 9.5 assessment, just over half of the ME children remained in the care of their biological mother. At age 9.5, a higher proportion of children in the ME group were being raised in families characterised by low SES and single parenthood than children in the comparison group. At the time of their 9.5-year assessment children in the ME group were slightly older than children in the non-ME comparison group. Finally, ME children’s mean IQ score was significantly lower than the non-ME children’s.

### Educational achievement at 9.5 years

#### Teacher ratings of achievement

At age 9.5 years, all of the comparison children and 95% of the ME children were attending mainstream primary (elementary) schools. As shown in Table 2, ME children were significantly more likely to be rated by their classroom teacher as being delayed compared to their non-ME peers in a range of subject areas. These spanned reading, mathematics, written and spoken language, health, art, and technology, with relative risks ranging from 2.8 to 3.9, and ORs from 3.4 to 7.6. The one subject exception was physical education, for which a similar proportion of ME and non-ME children were rated by their teachers as being delayed (OR = 1.97 [0.95–4.11]). Within the ME group, 78% (n = 65) were rated as delayed in at least one academic skill area, and 53% (n = 44) as delayed in three or more areas.

**Table 2:** Teacher ratings of children’s achievement across the school curriculum.

School curriculum domain	Methadone (*N* = 83) ^a^	Comparison (*N* = 97) ^b^	*p*	Odds ratio (95% CI) ^c^
% delayed reading	52	14	< .001	6.37 (3.13–12.98)
% delayed math	55	16	< .001	6.80 (3.37–13.70)
% delayed written language	65	20	< .001	7.64 (3.89–15.01)
% delayed expressive language	33	9	< .001	4.71 (2.07–10.76)
% delayed health	31	8	< .001	5.08 (2.15–11.98)
% delayed art	33	12	.001	3.42 (1.60–7.30)
% delayed technology	30	9	< .001	4.22 (1.84–9.67)
% delayed physical education	27	16	.07	1.97 (0.95–4.11)

^a^ One parent did not give permission to contact teacher.

^b^ Two non-ME children’s teachers failed to complete the questionnaire.

^c^ CI = confidence interval.

#### Standardized reading and mathematics achievement

Children’s performance on the WJ-III in the clinic setting is shown in Table 3. Consistent with their teacher-rated achievement at school, ME children obtained significantly lower standardized test scores than non-ME children. Their WJ-III reading and mathematics subtest scores, and their overall Broad Reading and Math scores, were between –0.72 and –1.03 SDs lower than non-ME children’s scores. For reading, ME children scored below non-ME children by 16.4 standard points in Letter-word Identification, 13.2 points in Reading Fluency, and 12.2 points in Passage Comprehension. For mathematics, ME children scored below non-ME children by 16.2 standard points in Calculation, 10.8 points in Math Fluency, and 13.5 points in Applied Problem Solving. These results indicate significant pervasive achievement gaps between ME and non-ME children, and collectively demonstrate that the ME children were, on average, almost 12-months behind the non-ME children in terms of reading and mathematics skill attainment.

**Table 3:** Performance on the Woodcock-Johnson Tests of Achievement (WJ-III).

WJ-III domain	Methadone (*N* = 84)	Comparison (*N* = 99)	*p*	*d* (95% CI) ^a^
*M (SD)* Broad Reading	87.74 (19.80)	104.35 (14.35)	< .001	0.97 (0.67–1.28)
*M (SD)* Letter-word ID	92.11 (19.33)	108.54 (14.24)	< .001	0.98 (0.67–1.29)
*M (SD)* Reading Fluency	87.56 (18.19)	100.76 (16.21)	< .001	0.77 (0.47–1.07)
*M (SD)* Passage Comp.	87.21 (15.60)	99.45 (9.60)	< .001	0.96 (0.65–1.27)
*M (SD)* Broad Math	86.80 (18.37)	103.03 (15.42)	< .001	0.96 (0.66–1.27)
*M (SD)* Calculation	82.14 (17.44)	98.31 (14.14)	< .001	1.03 (0.72–1.34)
*M (SD)* Math Fluency	86.85 (14.73)	97.65 (15.44)	< .001	0.72 (0.41–1.02)
*M (SD)* Applied Problems	93.06 (17.13)	106.53 (12.34)	< .001	0.91 (0.61–1.21)

^a^ *d* = Cohen’s *d* estimate of effect size,

CI = confidence interval.

#### Educational delay

Whilst the above results clearly suggest that children born to opioid-dependent mothers enrolled in MMT during pregnancy have, on average, lower achievement than non-ME children, they do not indicate the extent of serious educational delay amongst this vulnerable group. Table 4 shows the percentage of ME and non-ME children meeting criteria for an educational delay (a score –1 SD below the comparison group mean) in reading and/or mathematics on the WJ-III. As shown, ME children were 4 and 4.5 times more likely than non-ME children to be subject to reading (OR = 6.30 [2.94–13.47]) or mathematics delay (OR = 7.35 [3.36–16.07]). Overall, 57% vs. 15% were subject to any educational delay (i.e. reading and/or mathematics delay) (OR = 7.47 [3.71–15.02]).

**Table 4:** Educational delay on the WJ-III.

WJ-III domain	Methadone (*N* = 84)	Comparison (*N* = 99)	*p*	Odds ratio (95% CI) ^a^
% reading delay	44	11	< .001	6.30 (2.94–13.47)
% mathematics delay	45	10	< .001	7.35 (3.36–16.07)
% any educational delay	57	15	< .001	7.47 (3.71–15.02)

^a^ CI = confidence interval.

**Fig 1:**
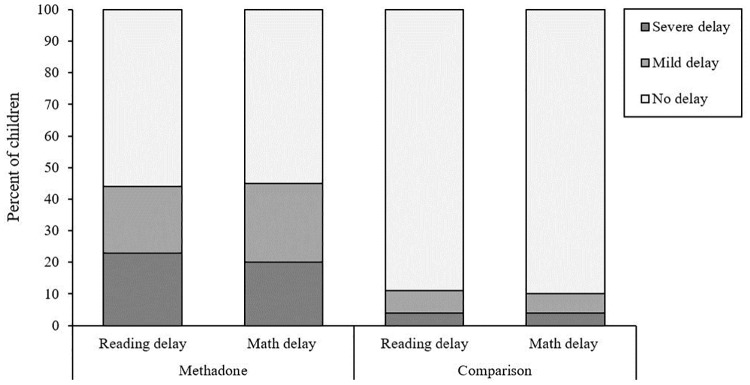
Rates of mild and severe reading and mathematics delay amongst methadone-exposed and comparison children.

Fig 1 shows children in each group classified according to the severity of their educational delay in reading and mathematics. ME children had significantly higher rates of both mild and severe reading delay on the WJ-III than non-ME children (21% vs. 7% and 23% vs. 4%, χ^2^ = 26.02, *p* < .001). They also had significantly higher rates of mild and severe mathematics delay (25% vs. 6% and 20% vs. 4%, χ^2^ = 29.04, *p* < .001).

**Fig 2:**
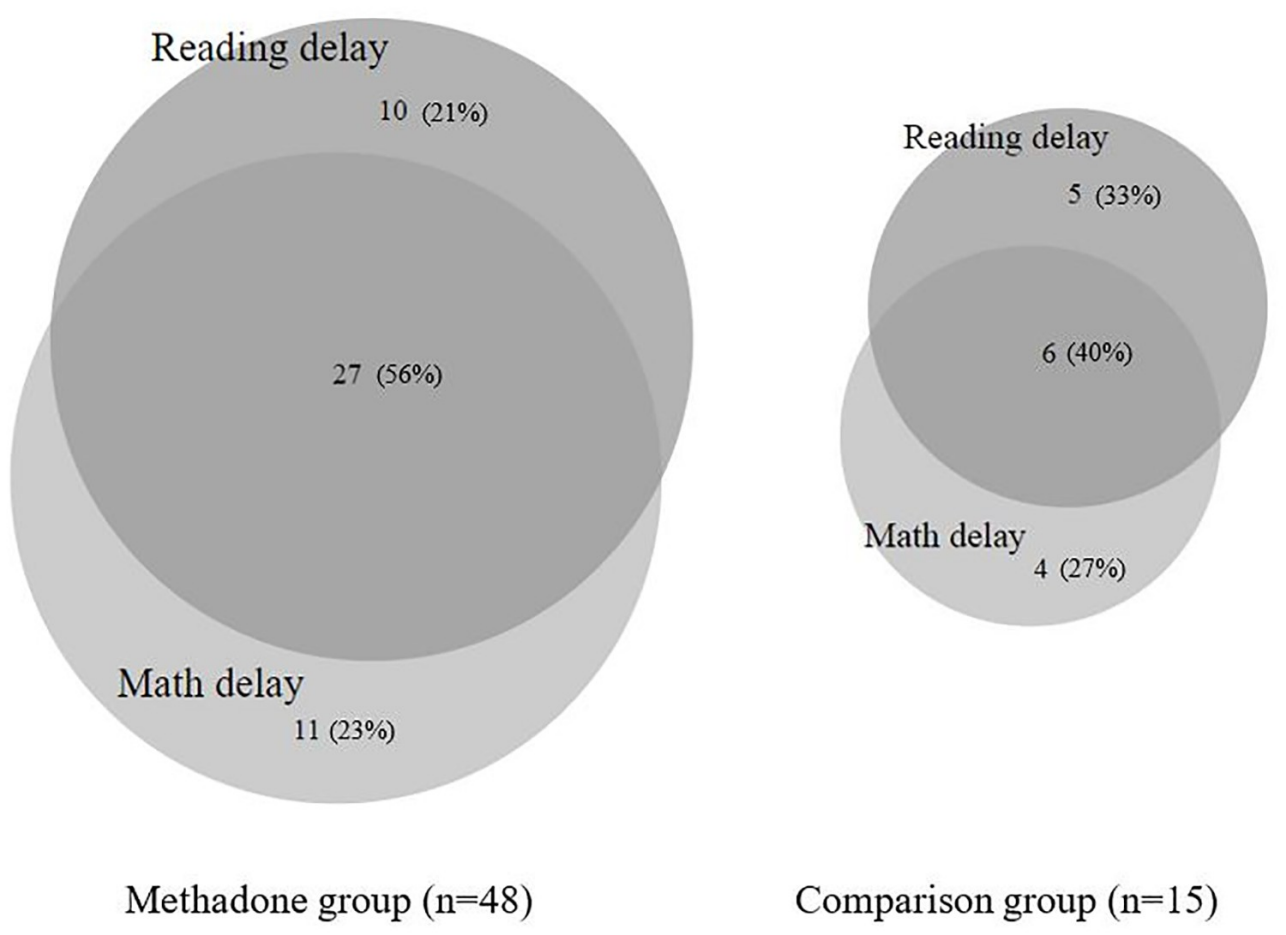
Patterns of co-occurring reading and mathematics delay amongst methadone-exposed and comparison children on the WJ-III.

Fig 2 shows the extent to which children in both study groups were at risk of significant delay in reading, mathematics, or both. As shown, co-occurring (reading and mathematics) delay was relatively common in both groups. Although, rates of co-occurring educational delay remained somewhat higher in the ME group, with more than half (56%) of the ME children with at least one affected educational domain subject to learning delays or difficulties in the other domain, compared to 40% of the non-ME children.

#### Special education

ME children were 4 times more likely than non-ME children to be receiving support from a special education service at school (36% vs. 9%, *p* < .001; OR = 5.56 [2.45–12.59]). In-class teacher aide assistance was the predominant special education support received by children in both groups.

#### Supplementary analyses

**Educational outcomes excluding children with a severe intellectual impairment (IQ ≤80)**. As demonstrated in S1 Table, when the rates of teacher-rated achievement delay were examined excluding children with a severe intellectual impairment, between-group differences were attenuated, but relative risks (2.3–3.1) and odds ratios (2.6–5.3) nonetheless remained significantly different.

As demonstrated in S2 Table, the effect sizes for the WJ-III reading and mathematics scores were also attenuated somewhat with the exclusion of children with a severe intellectual impairment. However, all between-group differences remained significant (*p* < .01), and the magnitude of these differences still ranged from moderate to large (–0.49 to –0.79 SDs).

Rates of SLD were also examined by calculating rates of WJ-III delay excluding children with a severe intellectual impairment. As shown in S3 Table, ME children’s risk for a reading SLD was 2.9 times greater than non-ME children’s (OR = 3.65 [1.62–8.23]). Their risk for a mathematics SLD was 3.2 times greater than non-ME children’s (OR = 4.06 [1.76–9.35]). ME children’s risk for having any SLD was 2.9 times greater than non-ME children’s, with 46% vs. 16% having a reading and/or a mathematics SLD (OR = 4.56 [2.19–9.48]). Similar to the results using the full sample, ME children had significantly higher rates of both mild and severe reading (21% vs. 7% and 11% vs. 4%, χ^2^ = 10.47, *p* = .005) and mathematics (24% vs. 6% and 8% vs. 4%, χ^2^ = 12.58, *p* = .002) SLDs than non-ME children (S1 Fig).

After the exclusion of children with a severe intellectual impairment, children in the ME group were still 3 times more likely to be receiving special education support (27% vs. 9%, *p* = .002; OR = 3.67 [1.53–8.79]).

**Sex differences**. An examination of sex differences in teacher ratings of delay across the school curriculum showed that teachers were more likely to rate boys as having delayed achievement across the domains of reading (OR = 2.82 [1.37–5.78], p = .005), written language (OR = 4.81 [2.30–10.08, p < .001), art (OR = 7.48 [2.89–19.32], p < .001), health (OR = 2.55 [1.10–5.89], p = .03), and technology (OR = 3.73 [1.55–8.99]), p = .003). There were no significant sex differences in teacher ratings of achievement in mathematics (OR = 1.84 [0.92–3.68], p = .08), spoken language (OR = 1.5 [0.68–3.23], p = .32), or physical education (OR = 1.25 [0.60–2.61], p = .55). One significant main effect of sex on WJ-III achievement was found. This was for the Reading Fluency subtest, and therefore the overall Broad Reading score, with girls in both groups obtaining higher scores than boys on this measure, *F*(1, 178) = 12.38, *p* = .001. Nonetheless, boys were no more likely than girls to be classified as having a WJ-III reading or mathematics delay. Boys were also found to be more likely than girls to have any special education involvement (OR = 6.01 [2.40–15.04]). No interaction effects between study group and sex were found.

#### Covariate analyses

The bivariate analyses performed thus far indicated that ME children had poorer educational outcomes relative to their non-ME peers. The aim of the next stage of the analysis was therefore to assess the extent to which prenatal methadone exposure predicted the likelihood of educational delay following adjustment for the confounding effects of other prenatal substance exposures, infant medical risks, and maternal social background factors measured at term. The variable “any educational delay”, representing either a WJ-III reading and/or mathematics delay was chosen as the dependent variable given that: a) the standardized measure was considered a more reliable achievement measure than the teacher’s ratings, and b) was chosen in place of the continuous WJ-III Broad Reading and Math scores given that pervasive delay was common.

As shown in Table 5 (step 2), being born to a mother maintained on methadone during pregnancy remained significantly associated with having an educational delay at age 9.5 following adjustment for confounding factors. However, this association was substantially attenuated and remained only marginally significant, with ME children’s odds of educational delay decreasing from 7.5 to 3.6 (*p* = .049). Other factors that were found to be associated with children’s increased odds for educational delay included maternal educational attainment level, (OR = 0.69, [0.50–0.89], p = .006), and any maternal use of benzodiazepines during pregnancy (OR = 2.70 [1.03–7.12], p = .044). The association between educational delay and any maternal use of alcohol during pregnancy was marginal (OR = 2.47 [0.99–6.15], p = .052).

**Table 5:** Summary of Logistic regression analysis for confounding factors associated with educational delay (0 = no delay, 1 = delay).

Variable	*B (SE)*	*p*	Odds ratio (95% CI)
**Step 1. Unadjusted**			
Methadone group status	2.01 (0.36)	< .001	7.47 (3.71–15.02)
**Step 2. Adjusted for confounding factors**			
Methadone group status	1.29 (0.65)	.049	3.62 (1.01–13.01)
Maternal educational underachievement	-0.40 (0.15)	.006	0.69 (0.50–0.89)
Prenatal benzodiazepine use	0.99 (0.49)	.044	2.70 (1.03–7.12)
Prenatal alcohol use	0.90 (0.47)	.052	2.47 (0.99–6.15)
Prenatal cigarette use	-0.51 (0.60)	.391	0.60 (0.19–1.93)
Prenatal cannabis use	-0.28 (0.49)	.571	0.76 (0.29–1.98)
Maternal depression score at term	-0.02 (0.03)	.626	0.99 (0.93–1.05)

## Discussion

This is the first prospective longitudinal study to examine the educational outcomes of 9.5-year-old children born to opioid-dependent mothers enrolled in MMT during pregnancy. We examined methadone-exposed and non-exposed children’s educational outcomes using a multi-method approach that combined ecologically based teacher report with independent standardized testing of children’s academic achievement and their risks of educational delay across the school curriculum. An additional issue of interest was whether ME children were at increased risk for poor educational outcomes when we excluded those children with severe intellectual impairment from our analysis. Further, we assessed the extent to which male ME children might perform less well on educational measures than their female peers. Finally, the extent to which the association between prenatal methadone exposure and children’s later risk of educational delay was potentially explained by a range of confounding factors was examined.

Findings showed that children born to mothers enrolled in MMT during pregnancy had poorer outcomes on all educational measures compared to a regional cohort of their non-ME peers. With the exception of physical education, teachers reported that children in the ME group were at much higher risk than non-ME comparison children of performing below average expected levels. That is, they were more than 12-months behind their same-age peers on each of the New Zealand curriculum domains of reading, mathematics, written and spoken language, art, and technology. The non-significant finding with respect to physical education may reflect that achievement in physical education typically does not require the literacy and numeracy skills that achievement in the other academic domains demands, particularly at lower levels of the curriculum [35].

In line with teacher observations, ME children were at increased risk of performing less well on the reading and mathematics subtests of the WJ-III Tests of Achievement. Specifically, they had, on average, acquired fewer reading skills including reading decoding, fluency and comprehension skills than non-exposed children of the same age from the same geographical region. Similarly, they had acquired fewer mathematics skills including basic calculation, mathematical fluency, and problem solving skills. The clinical importance of these findings was highlighted by the large magnitude of the group differences on each of the WJ-III subtests. In addition, ME children were at least 4 times more likely to be classified as having a reading and mathematics delay on that measure.

The current study findings are in accordance with those of two studies that have examined the educational achievement of children with prenatal opioid exposure. In a recent population data linkage study, it was found that children who were treated pharmacologically for NAS achieved significantly lower scores in school-based reading, writing, grammar, spelling and mathematics tests compared to demographically matched controls [29]. A history of NAS was associated with a two-fold increase in risk for having ever had delay on one of these achievement domains from age 9 to 15 years. Similarly, results from a smaller retrospective study showed that children born to, and raised by, heroin-addicted mothers performed more poorly on standardized reading and mathematics tests than comparison control children.

Extending these findings, we also examined the severity of children’s educational delay on the WJ-III. We assessed the proportion of children with mild (–1 SD to –2 SD) as well as severe (more than –2SDs) reading and mathematics delays. Even when using the more stringent cut-off score of more than –2SDs or lower relative to the comparison mean, ME children’s risk for a reading or mathematics severe delay was over 5 times that of the comparison group’s. Also important to note is that these rates of delay in the ME group may even be underestimated, due to the exclusion of other very high risk children, such as those born very preterm or with fetal alcohol syndrome, at the study outset.

It was also of interest to examine the proportion of children with difficulty or delay in both reading and mathematics, as these children may be at risk for a worse educational outlook than children with difficulty in only one, or neither, of these academic domains [30–32]. The high-risk nature of the ME group was further highlighted by their elevated rates of educational comorbidity (56% vs. 40%), particularly given that a much larger proportion of the ME group than the comparison group (children with at least one affected educational domain) was represented in this analysis.

Unsurprisingly, a larger proportion of ME children were enrolled in special education services at school, reflecting their greater educational and learning support needs. Importantly, our special education utilisation results also revealed that at least 21% of the ME group classified as having a WJ-III delay were reported as receiving no special education support. Clearly, the additional educational needs of a large proportion of the ME children are not currently being met.

We found that school-based and WJ-III delays were not confined to those ME children with severe intellectual impairment (IQ ≤80). Among children with an IQ >80, ME children were around 3 times more likely to be identified by their teachers as performing at below average or delayed levels across the seven curriculum domains listed above, and 3 times more likely to have special education involvement. They were also identified as showing greater difficulty on the WJ-III, with lower mean standardized reading and mathematics scores, and close to 3 times the risk of being classified as having a reading and/or a mathematics SLD. The finding that ME children with at least average intellectual ability were at increased risk for poor academic achievement could reflect their risk for decreased learning opportunities in the home, possible impacts of other specific cognitive difficulties such as language [1, 46, 47] or executive functioning difficulties [48, 49], self-regulatory behaviour problems [1, 50], or a combination of these early issues that compound or have cascading effects following the school transition [51, 52]. Further research is needed to identify the specific environmental and early childhood predictors of poor educational achievement in this population.

In the present study, teachers were more likely to rate ME and non-ME boys as having delayed achievement in reading, written language, health, art, and technology than girls in both groups. These differences were not found for mathematics, spoken language, or physical education. Boys were also more likely to have special education involvement at school than their female peers, and these findings are well supported in the literature. A male disadvantage is apparent across several functional areas of development, including school performance [1, 29]. Boys may be at increased risk for poorer teacher-rated achievement in some areas as they are more prone than girls to inattention, hyperactivity or disruptive behaviours that may hinder their opportunities to learn in the classroom, and, in turn, impact their academic performance [53, 54]. Boys’ relatively more disruptive classroom behaviour may also lead teachers to perceive boys’ achievement as below girls’, when in fact their achievement level may be the same as girls when measured through standardized testing. In the current study, the only sex difference found in children’s WJ-III scores was in Reading Fluency. Previous researchers have also reported that, on average, girls tend to achieve better in reading, but not mathematics, compared to boys. Our results showed that although boys were slower and less accurate readers than girls, they did however have similar sight word reading and reading comprehension skills. Speculatively, this might also be partly explained by the increased likelihood for boys to demonstrate attentional difficulties, with achievement scores in the timed Reading Fluency subtest depending somewhat on being continually on-task.

Understanding other factors associated with children’s increased risk for educational delay is critical to the identification of those most in need of early intervention and prevention strategies to improve their outcomes. In this study, we examined the extent to which being born to a methadone-maintained mother increased children’s risk for having any educational delay on the WJ-III adjusting for other factors correlated with maternal methadone use. Following adjustment for the effects of other prenatal substance exposures and maternal social risk (education level), the association between prenatal methadone exposure and children’s increased risk for educational delay was attenuated, yet remained significant. Lower maternal education level and maternal prenatal benzodiazepine use were also found to be significantly associated with children’s increased risk of having an educational delay.

The present study is the first to show that prenatal benzodiazepine exposure is independently associated with ME children’s educational delay. Previously, maternal use of benzodiazepines during pregnancy has been associated with poorer neonatal outcomes [55, 56], delayed cognitive development during infancy [57], and increased child behaviour problems [58, 59]. In addition, studies of prenatally opioid-exposed children have found independent associations between prenatal benzodiazepine exposure and poorer early inhibitory control [48] and increased maternal ratings of executive functioning problems [49]. It is possible that these associations are related to the greater psychiatric disturbance, particularly anxiety symptoms, experienced by women who take benzodiazepines during pregnancy [59]. This anxiety may lead to greater parenting difficulties, and contribute to a compromised rearing environment for the child. Further research is needed to understand the longer-term neurodevelopmental effects of prenatal exposure to benzodiazepines.

Maternal educational underachievement is common amongst prenatally substance-exposed children, and in turn, a lower maternal education level has been previously associated with their increased educational risk [29, 60, 61]. For example, Oei et al. (2017) found that among children who had been treated for NAS, having a parent with no educational qualification significantly increased their risk of middle childhood educational delay [29]. General population studies have also highlighted the importance of higher maternal educational attainment for children’s cognitive outcomes [62] even when accounting for other maternal psychosocial risks [63, 64]. Variation in maternal education is likely to differentiate the quality of ME children’s postnatal caregiving contexts, for example by impacting on the quality of the early learning environment and cognitive stimulation that parents can provide. This, in turn, is likely to impact children’s educational achievement.

There are limitations of this research that are important to note. First, despite our relatively large sample and high recruitment and retention, we may still have had limited power to detect other, smaller, significant effects in the multivariable analysis. For example, base rates of maternal alcohol use in our sample were relatively low, and this combined with our modest sample size may have precluded detecting a significant effect of maternal alcohol use on children’s educational delay.

Another limitation of the current study relates to the relative social advantage of the non-ME group compared to the ME group. The non-ME group was recruited at random, yielding a regionally representative comparison sample. This recruitment approach is helpful in terms of estimating the neurodevelopmental risk of the ME group compared to general population risks. There was a tendency for the non-ME comparison participants who were characterized by low maternal educational achievement to drop out of the study. However, this number was very small, and so it is unlikely that this had a large impact on the results.

Furthermore, we adjusted for maternal education in the final regression model to attempt to address this issue.

Another important issue concerns the examination of potential mediators of the association between prenatal methadone exposure and middle childhood educational delay. While methadone group status was associated with educational delay following covariate adjustment, maternal opioid-dependence and enrolment in MMT during pregnancy is likely to be a marker for increased socio-environmental adversities, including poverty, inadequate caregiving, and social service involvement [65, 66]. It has been proposed that the early biological vulnerability of substance-exposed children may be exacerbated or buffered from further risk, depending on the socio-environmental context in which they develop [33, 67]. Due to the increased risk for ME infants and children to grow up in environments that do not foster optimal learning and educational skill development, identifying potential modifiable factors (parenting, maternal mental health) to target in early intervention will be crucial to improving their educational outcomes. Research examining the environmental processes associated with ME children’s educational outcomes is necessary to aide our understanding of the mechanisms contributing to their risk, to help inform the development of effective interventions to support these children and families.

### Conclusion

The current findings indicate that many ME children do not achieve to the level of their non-ME peers at school or on independent standardized tests of educational achievement. The increased risk of educational delay for children in this study remained significantly associated with being born to a mother enrolled in MMT during pregnancy, even after a wide range of factors correlated with maternal opioid dependence were taken into account. These findings suggest that ME children should receive developmental follow-up from birth, with careful monitoring and assessment for early neurodevelopmental problems that may place them at elevated risk of academic difficulties. Monitoring and support across the transition to school is also likely to be important in ensuring early detection and intervention. In future, research should aim to determine which specific modifiable factors are associated with children’s educational delay. Implementing effective preventative intervention strategies for those children most at risk for a poor educational trajectory is crucial for supporting their optimal development and wellbeing, and minimizing their impacts on educational services.

## Supporting information

S1 Fig Rates of mild and severe reading and mathematics SLD amongst methadone-exposed and comparison children. (TIF)

S1 Table Teacher ratings of achievement across the school curriculum for children with IQ scores ≥ 80. (DOCX)

S2 Table Performance on the Woodcock-Johnson Tests of Achievement (WJ-III) for children with IQ scores ≥ 80. (DOCX)

S3 Table Specific learning delay on the WJ-III. (DOCX)
