# Supplementary figures and images for: Educational achievement at age 9.5 years of children born to mothers maintained on methadone during pregnancy
Source: PLoS One. 2019 Oct 10;14(10):e0223685. doi: 10.1371/journal.pone.0223685 (PMC6786534; doi:10.1371/journal.pone.0223685)

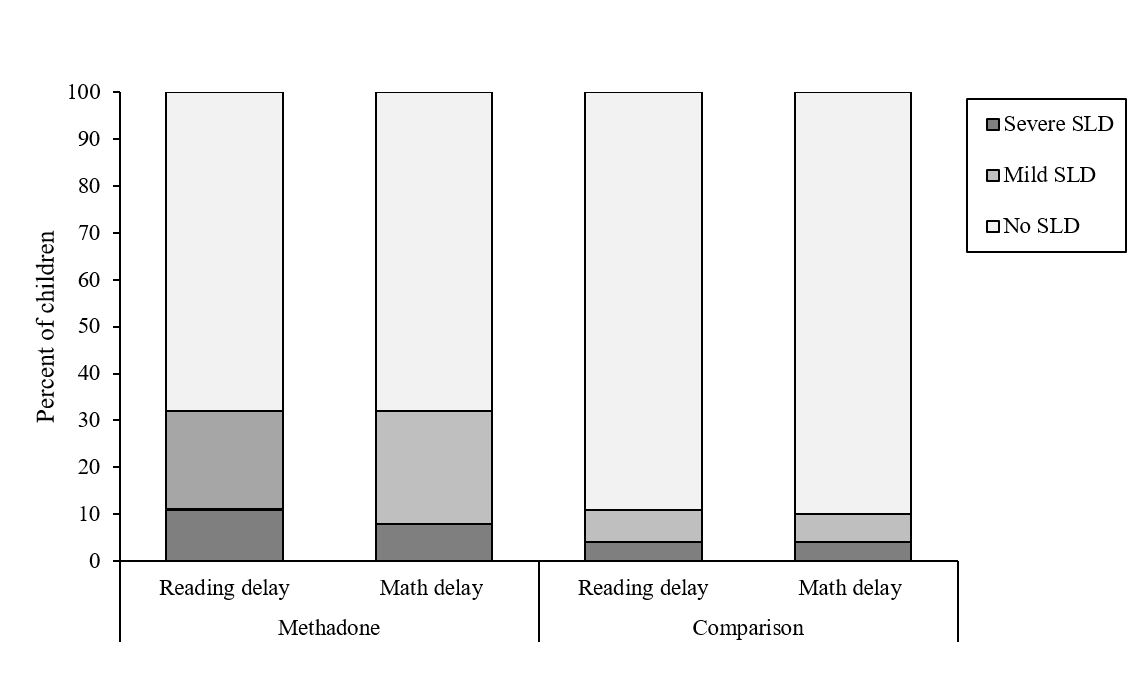

Supplement: S1 Fig — (TIF) [file pone.0223685.s001.tif]
