# Supplementary material for: Educational achievement at age 9.5 years of children born to mothers maintained on methadone during pregnancy
Source: PLoS One. 2019 Oct 10;14(10):e0223685. doi: 10.1371/journal.pone.0223685 (PMC6786534; doi:10.1371/journal.pone.0223685)
Supplement: S1 Table — (DOCX) [file pone.0223685.s002.docx]

| **Supplementary Table 1***.* **Teacher Ratings of Achievement across the School Curriculum for Children with IQ scores ≥ 80** | | | | |
| --- | --- | --- | --- | --- |
| **School curriculum domain** | **Methadone**  **(*N* = 65)** | **Comparison**  **(*N* = 95)** | ***p*** | **Odds ratio (95% CI) ^a^** |
| % delayed reading | 40 | 13 | <.001 | 4.61 (2.11 – 10.09) |
| % delayed math | 45 | 16 | <.001 | 4.30 (2.06 – 8.98) |
| % delayed written language | 55 | 19 | <.001 | 5.31 (2.61 – 10.79) |
| % delayed expressive language | 23 | 8 | .009 | 3.26 (1.29 – 8.24) |
| % delayed health | 23 | 8 | .009 | 3.26 (1.29 – 8.24) |
| % delayed art | 23 | 10 | .03 | 2.55 (1.07 – 6.11) |
| % delayed technology | 25 | 8 | .005 | 3.55 (1.42 – 8.89) |
| % delayed physical education | 23 | 16 | .25 | 1.60 (0.72 – 3.55) |
| ^a^ CI = confidence interval | | | | |
