# Supplementary material for: Educational achievement at age 9.5 years of children born to mothers maintained on methadone during pregnancy
Source: PLoS One. 2019 Oct 10;14(10):e0223685. doi: 10.1371/journal.pone.0223685 (PMC6786534; doi:10.1371/journal.pone.0223685)
Supplement: S2 Table — (DOCX) [file pone.0223685.s003.docx]

| **Supplementary Table 2***.* **Performance on the Woodcock-Johnson Tests of Achievement (WJ-III) for Children with IQ Scores ≥ 80** | | | | |
| --- | --- | --- | --- | --- |
| **WJ-III domain** | **Methadone**  **(*N* = 66)** | **Comparison**  **(*N* = 97)** | ***p*** | ***d* (95% CI) ^a^** |
| *M (SD)* Broad Reading | 93.55 (15.64) | 104.57 (14.42) | <.001 | 0.74 (0.42 – 1.06) |
| *M (SD)* Letter-word ID | 97.83 (15.21) | 108.77 (14.30) | <.001 | 0.75 (0.42 – 1.07) |
| *M (SD)* Reading Fluency | 92.27 (14.82) | 100.99 (16.30) | .001 | 0.56 (0.24 – 0.87) |
| *M (SD)* Passage Comp. | 91.76 (11.23) | 99.56 (9.60) | <.001 | 0.76 (0.43 – 1.08) |
| *M (SD)* Broad Math | 92.64 (13.87) | 103.14 (15.50) | <.001 | 0.71 (0.38 – 1.03) |
| *M (SD)* Calculation | 87.12 (14.25) | 98.34 (14.28) | <.001 | 0.79 (0.46 – 1.11) |
| *M (SD)* Math Fluency | 90.44 (12.40) | 97.51 (15.48) | .002 | 0.49 (0.18 – 0.81) |
| *M (SD)* Applied Problems | 98.44 (12.73) | 106.77 (12.29) | <.001 | 0.67 (0.34 – 0.99) |
| ^a^ *d* = Cohen’s *d* estimate of effect size, CI = confidence interval. | | | | |
