# Supplementary material for: Educational achievement at age 9.5 years of children born to mothers maintained on methadone during pregnancy
Source: PLoS One. 2019 Oct 10;14(10):e0223685. doi: 10.1371/journal.pone.0223685 (PMC6786534; doi:10.1371/journal.pone.0223685)
Supplement: S3 Table — (DOCX) [file pone.0223685.s004.docx]

| **Supplementary Table 3***.* **Specific Learning Delay on the WJ-III** | | | | |
| --- | --- | --- | --- | --- |
| **WJ-III domain** | **Methadone**  **(*N* = 66)** | **Comparison**  **(*N* = 97)** | ***p*** | **Odds ratio (95% CI) ^a^** |
| % reading SLD | 32 | 11 | .001 | 3.65 (1.62 – 8.23) |
| % mathematics SLD | 32 | 10 | .001 | 4.06 (1.76 – 9.35) |
| % any SLD | 46 | 16 | <.001 | 4.56 (2.19 – 9.48) |
| ^a^ CI = confidence interval. | | | | |
